# Personalized Surgical Decision-Making in Meniscal Tears: Short-Term Outcomes of Repair vs. Partial Meniscectomy in Mongolian Patients

**DOI:** 10.3390/jpm15120578

**Published:** 2025-11-28

**Authors:** Orgil Zorigtbaatar, Nomin-Erdene Minjuurdorj, Baatarjav Sosor, Gonchigsuren Dagvasumberel, Bayasgalan Gombojav, Naranbat Lkhagvasuren

**Affiliations:** 1 Department of Traumatology and Orthopedics, School of Medicine, Mongolian National University of Medical Sciences, Ulaanbaatar 14210, Mongolia; amd17f002@gt.mnums.edu.mn; 2 SODMED Mental Health Center, Ulaanbaatar 16062, Mongolia; amd21f005@gt.mnums.edu.mn; 3 National Trauma and Orthopedic Research Center, Ulaanbaatar 16092, Mongolia; baatarjav@ach.edu.mn; 4 Department of Radiology, Grandmed Hospital, Ulaanbaatar 17210, Mongolia; gonchigsuren@mnums.edu.mn; 5 Department of Epidemiology and Biostatistics, School of Public Health, Mongolian National University of Medical Sciences, Ulaanbaatar 14210, Mongolia

**Keywords:** meniscal repair, partial meniscectomy, personalized surgical decision-making, KOOS, Mongolian patients

## Abstract

**Objectives**: Arthroscopic meniscal surgery (AMS) is one of the most common orthopedic procedures worldwide, and its prevalence has been steadily increasing. In this study, we aimed to compare the short-term clinical outcomes (STCOs) and patient-reported outcome measures (PROMs) with anxiety and satisfaction in Mongolian patients. **Methods**: A prospective cohort study involved 103 patients who underwent arthroscopic knee surgery at The National Trauma Orthopedic Research Center and Grandmed Hospital in Mongolia between 2020 and 2023. STCO and PROM were calculated for the Knee Injury and Osteoarthritis Outcome Score (KOOS) subscores, the visual analog scale was assessed for pain (VAS), and Knee Range of Motion (ROM), Measures of Anxiety State-Trait Anxiety Inventory (STAI), and The Surgical Satisfaction Questionnaire (SSQ-8) were also used. **Results**: Out of 103 patients (69 for partial meniscectomy and 34 for meniscal repair), KOOSs improved significantly from pre-operative to post-operative levels. The Koos subscores for pain were 57.93 ± 12.58 pre-operatively and 80.93 ± 5.70 post-operatively; Koos subscores for Symptoms (KOOS Sx) were 54.13 ± 12.73, 80.27 ± 6.22; Koos subscores for Activities of Daily Living (KOOS ADL) were 61.28 ± 13.19, 79.61 ± 4.91; Koos subscores for Sports/Recreation (KOOS SR) were 42.28 ± 13.21, 72.04 ± 6.88; and Koos subscores for Quality of Life (KOOS QOL) were 45.08 ± 12.46, 77.85 ± 7.96. On the other hand, the pre-operative and post-operative results of the STAI were not significant (46.03 ± 8.2 vs. 39.59 ± 7.13, *p* = 0.781). **Conclusions**: In the present study, we elucidated patient- and injury-specific factors that may guide personalized surgical decision-making in Mongolian patients. Our findings suggest that AMS is a viable option for alleviating pain and enhancing function in the short term for patients with meniscal tears. The high PROMs and satisfaction scores reflect good-to-excellent results, and meniscal repair was associated with better outcome scores. While pre-operative anxiety levels were high, they decreased after surgery, although they did not entirely disappear.

## 1. Introduction

The meniscus is essential, acting as a shock absorber, a load distributor to reduce excessive contact pressure, and a secondary stabilizer of the knee, located at the periphery of the tibial surface in the knee [1,2,3]. It is susceptible to injury and has a limited ability to heal because it is an intra-articular structure with relatively low blood supply. Recently, there has been a growing trend in the surgical management of meniscal tears toward repairing the meniscus rather than performing a partial meniscectomy. This shift is due to the increasing recognition of the meniscus’s vital role in maintaining cartilage health and longevity [4,5,6,7,8]. On the other hand, partial meniscectomy is still a popular initial treatment option because it often leads to improved symptom relief, especially in the short term. Moreover, it has several advantages, including being technically easier to perform and requiring less operating time.

Numerous studies have investigated the radiologic outcomes of meniscectomy compared to meniscal repair, consistently revealing that the meniscectomy group experiences greater post-operative changes. However, while studies have compared the clinical outcomes, the results thus far remain inconclusive. Therefore, factors that affect the comparative outcomes between the two surgical techniques should be explored. For example, since osteoarthritis develops over time, the duration post-operation is a critical factor. As described by Kim et al., both the meniscectomy and the repair group showed improved patient-reported outcomes post-operatively (*p* < 0.001). However, the comparison of the radiologic outcomes between the two groups revealed that the repair group showed better radiologic outcomes than the meniscectomy group, despite limited healing of the repair site [9]. The results of arthroscopic partial meniscectomy were studied for 6–7 months [10], and lateral meniscal tears were reported to heal rapidly within 7 weeks, whereas medial meniscal tears healed within 5 weeks [11]. Another 5-year follow-up study revealed that refixation was more effective than partial meniscectomy in terms of clinical and radiologic outcomes and survival for at least 5 years’ follow-up. Refixation slowed the progression of arthritic changes, although it did not completely prevent arthrosis progression [12].

Furthermore, the patient’s anxiety and restlessness can lead to hesitancy and avoidance during surgery, post-operative complications, joint function improvement, and healing [13]. The State Anxiety Scale (S-Anxiety) is used in many fields, including surgery, to assess whether a patient is anxious, and a score of 40 or higher is considered anxious. Few studies have examined the effects of the aforementioned factors (period of follow-up and presence of concomitant ACL reconstruction) on the comparative clinical outcomes of meniscectomy versus repair.

Arthroscopic surgery of the knee joint has been around for over 60 years and has since become a common procedure in many countries. Meniscectomy was first introduced to the clinic in 1962 by Japanese surgeon Masaki Watanabe [14], and further development and meniscal repair were pioneered by Shahriaree H. and Richard L. O’Connor [15], who also collaborated on the development of high-powered scopes. Since 1964, Canadian physician Robert Jackson has devoted much of his time to developing, teaching, and promoting arthroscopic surgery, first in the Western world [16] and then in North America [17], and was named one of *Sports Illustrated’s* forty most influential people in sports history, the only physician on the list.

Since then, partial meniscectomy has become the most widely performed arthroscopic surgical procedure worldwide, with an estimated 61 per 100,000 knee meniscectomies performed worldwide [17], and approximately 700,000 meniscectomies are performed in the United States each year [18]. However, AMS has been steadily adopted in Mongolia over the past two decades but remains understudied at a national level. Mongolia is a lower–middle-income country with a population of 3.5 million, and the National Trauma and Orthopedic Research Center of Mongolia (NTAORCM) is the only state-owned specialized center serving all cities and districts in Mongolia [19]. This procedure was first introduced at the National Trauma and Orthopedic Research Center of Mongolia (NTAORCM) with support from French surgeons in 2000 and has been performed intensively since 2012. Since 2014, Grandmed Hospital has also offered AMS. Local service reports indicate that arthroscopic partial meniscectomy of the knee was performed in approximately 118 patients over five years (2015–2019) nationwide, and between 2014 and 2019, a total of 57 patients underwent arthroscopic meniscectomy and/or repair at Grandmed Private Hospital (institutional conference proceedings). Despite this growth, Mongolia lacks peer-reviewed, prospective outcome studies comparing meniscal repair with partial meniscectomy. This gap provided a strong rationale for the present prospective cohort study, designed to quantify short-term clinical outcomes and patient-reported outcome measures (PROMs), including anxiety and surgical satisfaction, in Mongolian patients with isolated meniscal tears. That is why this is the first study conducted in Mongolia. Therefore, in the present study, we compared the short-term clinical outcomes of partial meniscectomy and meniscal repair and elucidated the factors that can guide personalized surgical decision-making in Mongolian patients.

## 2. Materials and Methods

### 2.1. Study Design and Setting

We conducted a prospective cohort study at two tertiary centers in Ulaanbaatar (National Trauma Orthopedic Research Center in Mongolia and Grandmed Hospital) from July 2020 to April 2023. Consecutive eligible patients undergoing AMS for meniscal tears were invited to participate.

### 2.2. Participants and Eligibility

Inclusion criteria were: (1) age ≥ 18 years; (2) arthroscopically treated meniscal tear (ICD-10 S83.2, M23.2; procedures coded ICD-9 80.6, 81.47); (3) isolated meniscal lesion without concomitant ligament injury; and (4) availability for 1-year follow-up. Exclusion criteria included prior ipsilateral knee arthroplasty, fracture around the knee, and advanced radiographic osteoarthritic change (Outerbridge grade 3–4) incompatible with meniscal preservation. We evaluated 106 knees from 103 patients (three bilateral cases).

### 2.3. Diagnostic Workflow and Treatment Allocation

All patients underwent standardized clinical assessments (history, physical examination, and standing radiographs) and confirmatory MRI before surgery. The final diagnosis, tear pattern, and location, and treatment (partial meniscectomy vs. repair) were determined intraoperatively by the attending arthroscopic surgeon based on tear morphology, vascular zone, tissue quality, and knee stability. Repair was favored for repairable tears in vascularized zones; partial meniscectomy was chosen for non-repairable tears with poor tissue quality or in avascular regions.

### 2.4. Assessment Battery and Timing

Data were collected using a structured case report form totaling 131 items:

- From medical history/records (61 items): Demographic data (12 items); Standardized knee examination items captured from the AMIS Technology Application Manager platform (16 items); Physical exam and imaging findings, including special tests and MRI/radiographic details (33 items).
- Patient-reported measures (70 items): KOOS (42 items; completion time ~15 min); State-Trait Anxiety Inventory, Form Y-1 (STAI; 20 items; ~9 min); Surgical Satisfaction Questionnaire (SSQ-8; 8 items; ~3 min)

### 2.5. Outcome Definitions and Follow-Up Schedule

Primary short-term outcomes were assessed at baseline (pre-operative) and at 1 year post-operatively: KOOS subscales (Pain, Symptoms, ADL, Sports/Recreation, QOL), VAS for pain, knee range of motion (ROM), and SSQ-8. Early post-operative trajectories were characterized using VAS and ROM at post-operative day 1 and days 3–7. Anxiety was assessed with STAI pre-operatively and at days 3–7.

### 2.6. Data Quality and Handling

All CRFs were checked for completeness at the point of care. Prior to analysis, continuous variables were screened for outliers and missingness; implausible values were re-checked against source records. Bilateral cases were recorded at the knee level with patient-level linkage.

### 2.7. Statistical Analysis

Group differences in age and BMI were compared using independent *t*-tests. Longitudinal effects of time (pre-op, early post-op, 1-year), treatment type (meniscectomy vs. repair), and their interaction were modeled using mixed two-way ANOVA with Greenhouse–Geisser correction for sphericity. Where appropriate, within-subject changes were evaluated using paired *t*-tests with Bonferroni-type correction (α = 0.05/3 = 0.017). Significance was set at *p* < 0.05 (two-sided). Analyses were performed in RStudio (R 3.3.0).

### 2.8. Ethics

The study was approved by the Research Ethics Committee of the Mongolian National University of Medical Sciences (approval No. 2020/3-5; date of 12 June 2020). All participants provided written informed consent.

## 3. Results

Out of 103 patients, the average age was 34.4 ± 8.6 years (range: 18–48 years); male participants comprised 61.2% (n = 63), and females 38.8% (n = 40). Of the total 106 tears, 60.3% (n = 64) were found in males, while 39.6% (n = 42) occurred in females. The main causes of tears were sports injuries and household injuries (Table 1).

Table 2 shows the location and types of meniscal tears. In the meniscectomy group, 59 patients had medial tears, and 8 patients had lateral tears. Meanwhile, in the meniscal repair group, 31 patients had medial tears, and 2 patients had lateral tears.

The meniscal surgery outcomes were presented in Table 3. Knee Injury and Osteoarthritis Outcome Scores (KOOS) improved significantly from pre-operative to post-operative levels. Koos subscores for Pain (KOOS Pain) pre-operatively were 57.93 ± 12.58, post-operatively were 80.93 ± 5.70; Koos subscores for Symptoms (KOOS Sx) 54.13 ± 12.73, 80.27 ± 6.22; Koos subscores for Activities of Daily Living (KOOS ADL) 61.28 ± 13.19, 79.61 ± 4.91; Koos subscores for Sports/Recreation (KOOS SR) 42.28 ± 13.21, 72.04 ± 6.88; Koos subscores for Quality of Life (KOOS QOL) 45.08 ± 12.46, 77.85 ± 7.96. The female had significantly lower pre-operative subscores despite KOOS Sx (*p* = 0.194) and lower Post-operative subscores for Pain (KOOS Pain) (*p* = 0.268) and for Symptoms (KOOS Sx) (*p* = 0.458). In the two-way mixed ANOVA results, the VAS score appeared to be reduced throughout the treatment period in the Meniscectomy group, with a mean value at post-operative 1 year of 5.48-fold lower (0.13 ± 0.38) than at pre-operative (5.61 ± 0.75) (*p* = 0.036). On the other hand, the VAS score of the meniscal repair group showed a post-operative reduction to 0.18 ± 0.46. A mixed two-way ANOVA analysis in the Meniscectomy group also revealed that the VAS score was significantly improved at 3–7 days post-operatively compared to the pre-operative score (*p* = 0.018). A similar trend was also observed for KOOS symptoms when the post-operative 1-year score was compared to the pre-operative score (*p* = 0.036). Furthermore, when KOOS Quality of Life scores were compared, the 1-year post-operative score was also significantly improved compared to the pre-operative score (*p* = 0.011). For the Meniscal Repair group, the VAS score at 1 year post-operatively was significantly lower than the pre-operative score (*p* = 0.041), and the ROM at 1 year post-operatively was higher than the pre-operative ROM (*p* = 0.001).

Furthermore, the meniscal surgery outcomes, in terms of satisfaction and anxiety, are displayed in Table 4. There were no significant differences between the two groups. However, the mixed ANOVA analysis showed that the 3–7 days post-operative index for STAI was significantly decreased from the baseline (*p* = 0.029) in the Meniscectomy group (Table 4).

## 4. Discussion

Arthroscopic meniscus repair is an outpatient surgical procedure used to repair torn cartilage in the knee. The torn meniscus is repaired with various minimally invasive techniques and needs post-operative care to aid healing. Physical therapy plays a key role in restoring full knee function, which usually occurs 4–5 months after surgery. However, a meniscus tear requires a blood supply to heal, but only the outer third of the meniscus has the blood supply for healing; therefore, repairs are generally limited to this outer region of the meniscus.

Meniscus tears can be treated by removal (meniscectomy), repair, or, in rare cases, replacement. The primary goal of surgery is to preserve the healthy meniscus, so repair is attempted when the tear is amenable to repair. Numerous studies have shown that meniscectomy, which involves removing the damaged tissue, yields good short-term results but may lead to the development of arthritis over the next ten to twenty years [20,21,22,23]. In contrast, meniscus repair tends to have positive outcomes, although recovery is longer than with meniscectomy and is only suitable for repairable tears. In the study by Gabr et al., after a 53-month follow-up, arthroscopic repair in the pediatric population proved to be an effective treatment with a low failure rate and good post-operative clinical results, with the added benefit of preserving meniscal tissue [23]. Another study by Khan et al. indicated that, among 92 patients who underwent meniscal repair or partial meniscectomy, meniscal repair should be favored over partial meniscectomy when surgically treating meniscal tears [24]. As mentioned earlier, studies reporting differences in the clinical outcomes of meniscectomy versus repair have yielded varied results. A retrospective study by Lee et al. demonstrated that both techniques had good outcomes. IKDC scores improved from 46.6 to 81.7 after meniscectomy and from 45.9 to 84.4 after repair (*p* < 0.001); however, meniscal repair had a better prognosis in the long run [25]. The 3-year outcome retrospective cohort study demonstrates that meniscal repair is more effective than meniscectomy in improving patient outcomes, with a higher proportion of patients achieving excellent and good results and higher scores on the Lysholm scale [26].

The key finding of this study is that meniscal repair outcomes have shown better scores compared to meniscectomy. The Knee Injury and Osteoarthritis Outcome Score (KOOS) subscores demonstrated significant improvements from pre-operative to post-operative levels. The pre-operative Pain subscore was 57.93 ± 12.58, increasing to 80.93 ± 5.70 after surgery. Additionally, the subscores for Symptoms improved from 54.13 ± 12.73 to 80.27 ± 6.22; the subscores for Activities of Daily Living rose from 61.28 ± 13.19 to 79.61 ± 4.91; the subscores for Sports/Recreation increased from 42.28 ± 13.21 to 72.04 ± 6.88; and those for Quality of Life increased from 45.08 ± 12.46 to 77.85 ± 7.96. However, we did not observe significant differences in the VAS scores between the two groups. As described by Ntagiopoulos et al., KOOSs at nine-year follow-up in older patients after partial meniscectomy showed worsening symptoms, which were related to increased age, higher BMI, and delayed diagnosis [27]. On the other hand, a meta-analysis comparing the effectiveness of meniscectomy and meniscus repair revealed no difference in changes to the KOOS subscales, specifically the KOOS pain subscale [28]. Patients who underwent partial meniscectomy with needle or standard technique also showed that adopting a needle arthroscopic technique for partial meniscectomy was associated with significantly improved VAS and KOOS pain scores two weeks post-operatively. However, the differences observed were no longer present six weeks after the surgery [29]. Large-scale meta-analyses have shown that meniscal repair is associated with a lower risk of developing knee osteoarthritis compared to partial meniscectomy. Consistent with these findings, the meniscal repair group in our study demonstrated higher KOOSs at one-year follow-up, suggesting that long-term observation may reveal a reduced risk of osteoarthritis in this group. These results highlight the potential long-term benefits of meniscal preservation in improving patient outcomes and preventing degenerative changes [30].

Our present study is a prospective cohort design, which introduces the potential for selection bias since patients were not randomly assigned to treatment groups. However, this concern is somewhat mitigated as both groups (meniscectomy and meniscal repair) show minimal differences in demographics and in the characteristics of meniscal and cartilaginous injuries. A limitation of our study is that only 103 participants were included, due to the recent introduction of arthroscopic partial meniscectomy in our country. Additionally, as this study only covers short-term clinical outcomes, future research should include extended follow-up beyond one year to assess the durability of patient-reported outcome measure (PROM) gains, structural cartilage changes, and radiographic/MRI progression following repair compared to meniscectomy. Furthermore, it is important to investigate the impact of baseline and perioperative anxiety (using the State-Trait Anxiety Inventory, STAI) as well as other psychosocial factors on recovery, adherence, and patient satisfaction. Future studies should also test brief interventions aimed at reducing anxiety. Finally, there is an urgent need to develop a comprehensive knee arthroscopy registry in Mongolia. This registry should meticulously capture a wide range of data, including clinical indications, surgical techniques, types of implants, complication rates, details of re-interventions, and patient-centered outcomes. This would enhance our understanding, improve surgical practices, and elevate the standard of care provided to patients.

This study is the first to evaluate the outcomes of meniscal repair and partial meniscectomy in Mongolian patients. Despite certain limitations, the findings provide valuable baseline data that can guide evidence-based, personalized surgical decision-making for this population and address the existing data gap regarding regional comparisons in managing meniscal injuries. Our results suggest that meniscal preservation should be prioritized whenever repair is feasible, as patients show greater improvements in KOOS subscale scores at one year compared to those who underwent partial meniscectomy, while noting that pain relief (measured by VAS) is similar in both groups. These data can serve several purposes: (i) inform shared decision-making by quantifying expected short-term benefits, (ii) guide service planning for AMS capacity and training focused on repair techniques, (iii) encourage standardized PROM collection (KOOS, SSQ-8, STAI) in routine care, and (iv) motivate integration of brief perioperative anxiety screening and counseling, given the observed decrease in STAI scores post-operatively. In resource-constrained settings, prioritizing repairs for tears that can be fixed may enhance patient function and quality of life while potentially reducing long-term degenerative changes.

## 5. Conclusions

Meniscectomy has long been regarded as the standard treatment for meniscal injuries. However, with advancements in arthroscopic repair techniques and a deeper understanding of the meniscus’s role in maintaining knee biomechanics, the surgical trend has shifted toward meniscal preservation whenever possible.

Our results demonstrated that meniscal repair achieved significantly better KOOS sub-scores across all domains compared with partial meniscectomy, indicating superior short-term functional outcomes. These findings emphasize the importance of preserving the native meniscus when repair is feasible, while long-term follow-up studies are needed to confirm its joint-protective benefits in the Mongolian population. Moreover, our study supports the use of patient-specific strategies to optimize functional recovery after meniscal injury.

## Figures and Tables

**Table 1:** General Characteristics of the Study Participants.

Variables	Meniscectomy	Meniscal Repair	Total	*p*-Value
	n = 69	n = 34	n = 103	
	Mean ± SD	Mean ± SD	Mean ± SD	
Age (years)	35.30 ± 8.57	32.65 ± 8.36	34.43 ± 8.56	0.137
BMI, kg/m^2^	26.78 ± 4.15	25.64 ± 3.86	26.40 ± 4.07	0.173
Gender	N (%)	N (%)	N (%)	
Male	42 (0.61)	21 (0.62)	63 (0.61)	0.999
Female	27 (0.39)	13 (0.38)	40 (0.39)	
Education				
None	1 (0.01)	-	1 (0.01)	
High School	12 (0.17)	5 (0.15)	17 (0.17)	
Vocational training	10 (0.15)	8 (0.24)	18 (0.15)	
Higher education	46 (0.67)	21 (0.61)	67 (0.67)	
Marital status				
Married	57 (0.83)	25 (0.74)	82 (0.83)	0.415
Single and others	12 (0.17)	9 (0.26)	21 (0.17)	
Employment status				
Employed	59 (0.85)	29 (0.85)	88 (0.85)	0.999
Unemployed	10 (0.15)	5 (0.15)	15 (0.15)	
Ethnicity				
Khalkh	63 (0.91)	32 (0.94)	95 (0.92)	0.912
Others	6 (0.09)	2 (0.06)	8 (0.08)	
Causes of tears				
Sport Injury	27 (0.39)	19 (0.56)	46 (0.45)	
Household injury	24 (0.35)	11 (0.32)	35 (0.34)	
Auto Accident	3 (0.04)	-	3 (0.03)	
Others	6 (0.09)	-	4 (0.06)	
Cause unknown	9 (0.13)	4 (0.12)	13 (0.12)	
BMI classification				
Normal (<24.9)	24 (0.35)	17 (0.50)	41 (0.40)	0.534
Overweight (25.0–29.9)	31 (0.45)	13 (0.38)	44 (0.43)	
Obese (≥30.0)	14 (0.20)	4 (0.12)	18 (0.17)	

Data are expressed as mean ± SD and number percentage (%). Abbreviations: SD, standard deviation; BMI, body mass index.

**Table 2:** Location and Types of Meniscal Tears.

Variables	Meniscectomy	Meniscal Repair	Total	*p*-Value
	n = 69	n = 34	n = 103	
	Mean ± SD	Mean ± SD	Mean ± SD	
Symptom duration, months	19.24 ± 37.58	29.35 ± 54.14	22.58 ± 43.74	0.333
Duration of surgery, minutes	60.65 ± 31.71	83.53 ± 25.21	68.20 ± 31.51	0.000
Location	N (%)	N (%)	N (%)	
Medial	59 (0.86)	31 (0.91)	90 (0.87)	0.654
Lateral	8 (0.12)	2 (0.06)	10 (0.10)	
Both	2 (0.02)	1 (0.03)	3 (0.03)	
Tear pattern				
Radial/Transverse	25 (0.36)	12 (0.35)	37 (0.36)	
Oblique	6 (0.09)	3 (0.09)	9 (0.09)	
Vertical/Longitudinal	12 (0.17)	6 (0.18)	18 (0.17)	
Horizontal	10 (0.14)	4 (0.12)	14 (0.13)	
Complex/Degenerative	2 (0.03)	-	2 (0.02)	
Bucket-Handle	5 (0.08)	5 (0.15)	10 (0.10)	
Discoid Meniscus	1 (0.01)	1 (0.03)	2 (0.02)	
Radial/Longitudinal	2 (0.03)	-	2 (0.02)	
Other	6 (0.09)	3 (0.09)	9 (0.09)	

Data are expressed as mean ± standard deviation and as numbers and percentages (%).

**Table 3:** Meniscal Surgery Outcome.

Variables	Meniscectomy ^a,b,c^	Meniscal Repair ^d,e^	Total	*p*-Value
	n = 69	n = 34	n = 103	
	Mean ± SD	Mean ± SD	Mean ± SD	
VAS Score				
Pre-operative	5.61 ± 0.75	5.47 ± 0.89	5.56 ± 0.80	0.442
Post-operative, 1 day	2.51 ± 0.72	2.47 ± 0.96	2.50 ± 0.80	0.845
Post-operative, 3–7 days	0.88 ± 0.65	0.76 ± 0.70	0.84 ± 0.67	0.408
Post-operative, 1 year	0.13 ± 0.38	0.18 ± 0.46	0.15 ± 0.40	0.615
ROM				
Pre-operative	94.49 ± 15.77	95.29 ±16.19	94.76 ± 15.83	0.812
Post-operative, 1 day	110.72 ± 8.96	108.82 ± 8.80	110.09 ± 8.91	0.309
Post-operative, 1 year	129.13 ± 3.32	128.53 ± 5.00	128.93 ± 3.94	0.528
KOOS Pain				
Pre-operative	57.09 ± 13.39	59.64 ± 10.73	57.93 ± 12.58	0.299
Post-operative, 1 year	79.95 ± 5.28	82.92 ± 6.08	80.93 ± 5.70	0.018
KOOS Symptoms				
Pre-operative	52.48 ± 13.11	57.46 ± 11.37	54.13 ± 12.73	0.051
Post-operative, 1 year	79.04 ± 5.78	82.77 ± 6.43	80.27 ± 6.22	0.006
KOOS Activities of Daily Living				
Pre-operative	59.53 ± 13.74	64.84 ± 11.39	61.28 ± 13.19	0.041
Post-operative, 1 year	78.37 ± 4.24	82.14 ± 5.26	79.61 ± 4.91	0.001
KOOS Sports/Recreation				
Pre-operative	40.80 ± 13.60	45.29 ± 11.99	42.28 ± 13.21	0.091
Post-operative, 1 year	71.38 ± 5.62	73.38 ± 8.85	72.04 ± 6.88	0.234
KOOS Quality of Life				
Pre-operative	43.21 ± 11.83	48.90 ± 13.01	45.08 ± 12.46	0.035
Post-operative, 1 year	76.63 ± 8.07	80.33 ± 7.24	77.85 ± 7.96	0.022

Data are expressed as mean ± SD. Abbreviations: KOOS, Knee Injury and Osteoarthritis Outcome Score; VAS, Visual Analog Scale; ROM, Range of Motion. Mixed two-way ANOVA: ^a^ pre-operative vs. post-operative, 3–7 days, *p* = 0.018 in VAS score; ^b^ pre-operative vs. post-operative, 1 year, *p* = 0.036 in KOOS symptoms; ^c^ pre-operative vs. post-operative, 1 year, *p* = 0.011 in KOOS quality of life. ^d^ pre-operative vs. post-operative, 1 year, *p* = 0.041 in VAS score; ^e^ pre-operative vs. post-operative, 1 year, *p* = 0.001 in ROM.

**Table 4:** Meniscal Surgery Outcome by Satisfaction and Anxiety.

Variables	Meniscectomy ^a^	Meniscal Repair ^b^	Total	*p*-Value
	n = 69	n = 34	n = 103	
	Mean ± SD	Mean ± SD	Mean ± SD	
STAI				
Pre-operative	46.19 ± 8.31	45.71 ± 8.23	46.03 ± 8.20	0.781
Post-operative, 3–7 days	40.13 ± 6.84	38.50 ± 7.66	39.59 ± 7.13	0.298
SSQ-8				
Post-operative, 1 year	3.97 ± 0.74	3.83 ± 0.61	3.93 ± 0.69	0.298

Data are expressed as mean ± SD. Abbreviations: STAI, State-Trait Anxiety Inventory; SSQ-8, Surgical Satisfaction Questionnaire. Mixed two-way ANOVA: ^a^ pre-operative vs. post-operative, 3–7 days, *p* = 0.029 in STAI, in Meniscectomy; ^b^ pre-operative vs. post-operative, 1 year, *p* = 0.055 in STAI.

## Data Availability

The datasets used and/or analyzed during the current study are available from the corresponding authors on reasonable request.
